# Evaluation of the linear and volumetric measuring changes in different positions in CBCT

**DOI:** 10.1002/cre2.732

**Published:** 2023-04-04

**Authors:** Samira Fani, Ehsan Moudi, Sina Haghanifar, Seyedali Seyedmajidi, Arash Poursattar‐Bejeh Mir

**Affiliations:** ^1^ School of Dentistry Babol University of Medical Science Babol Iran; ^2^ School of Dentistry, Oral Health Research Center, Health Research Institute Babol University of Medical Sciences Babol Iran; ^3^ School of Dentistry, Dental Materials Research Center, Health Research Institute Babol University of Medical Science Babol Iran; ^4^ Dental Materials Research Center, Health Research Institute Babol University of Medical Science Babol Iran

**Keywords:** cone‐beam computed tomography, dimensional measurement accuracy, software

## Abstract

**Objectives:**

The effect of head position on reproducibility of volumetric measurements on Cone‐beam computed tomography (CBCT) scans has not been evaluated before. Thus, this study aimed to assess the changes in linear and volumetric measurements on CBCT scans in different positions of the respective object.

**Materials and methods:**

In this experimental study, 16 balls were placed in containers and underwent CBCT in neutral position and 8 different altered positions. The horizontal and vertical diameters of each ball were measured by OnDemand software while the volume of each ball was quantified by ITK‐Snap software. The reproducibility of linear and volumetric measurements in nine different positions was analyzed by the Cronbach's *α*. Paired *t*‐test was applied to analyze the difference in volumetric and linear measurements of the balls in the anterior and posterior halves of the scans in neutral position versus the upward and downward tilts, and right and left halves of the scans in neutral position versus the right and left tilts.

**Results:**

The Cronbach's *α* was found to be .982 and .933 for volumetric and linear measurements, respectively, indicating high reproducibility of the measurements. No significant difference was noted in the mean linear measurements on CBCT scans at different positions compared with neutral position. In volumetric measurements, maximum difference between neutral and the other positions was less than 1%.

**Conclusions:**

CBCT is a reliable modality for linear and volumetric measurements in different positions.

## 1 INTRODUCTION

Since the advent of X‐ray and its application for imaging in medicine, researchers have attempted to improve the imaging technology and enhance image quality and dimensional accuracy of the radiographs. X‐ray imaging of the oral and maxillofacial region is highly important for assessment of dental structures. The conventional radiographic modalities have limitations such as image distortion, superimposition of different structures, and inaccurate visualization of the respective structures (Panjnoush et al., 2017).

Optimal image quality has a fundamental role in correct diagnosis and treatment planning in dental practice. The conventional computed tomography is an accurate modality for dimensional measurements. However, it has a high cost and high patient radiation dose, which limit its applications in dentistry. Due to the use of minimum voxel size in the head and neck region Cone‐beam computed tomography (CBCT) is a valuable imaging modality for diagnosis, treatment planning and evaluation of treatment outcome in dental practice, especially in oral and maxillofacial surgery and dental implant treatment planning. CBCT has advantages such as lower patient radiation dose than the conventional computed tomography, and enabling accurate and reproducible dimensional measurements (Almeida et al., 2021; Mupparapu et al., 2021).

CBCT is increasingly used for clinical applications such as identifying the exact location of pathological lesions, dental implant treatment, linear measurements before implant placement, assessment of temporomandibular joints, orthodontic analysis, airway analysis, and fabrication of surgical guides. Such clinical procedures require CBCT scans with adequately high geometric accuracy to achieve satisfactory results (García‐Sanz et al., 2017).

A number of factors such as field of view, radiation quality and quantity, voxel size, and rotational arc affect the quality and accuracy of CBCT images, and can influence image properties such as noise, contrast, resolution, and artifacts (Panjnoush et al., 2017).

Several studies have evaluated the accuracy of linear and volumetric measurements made on CBCT scans, yielding controversial results (Dong et al., 2019; Etemadi Sh et al., 2021; García‐Sanz et al., 2017; Panjnoush et al., 2017; Rokn et al., 2016; Sabban et al., 2015; Sönmez et al., 2018; Tofangchiha et al., 2016; Zhou et al., 2015). In addition to the accuracy of linear and volumetric measurements, the reproducibility of such measurements in different positions is also important. Risk of incorrect patient position is high in extraoral radiography, and the effect of head position on reproducibility of volumetric measurements has not been evaluated before. Thus, this study aimed to assess the changes in linear and volumetric measurements on CBCT scans taken in different positions of the respective object.

## 2 MATERIALS AND METHODS

**Figure 1:**
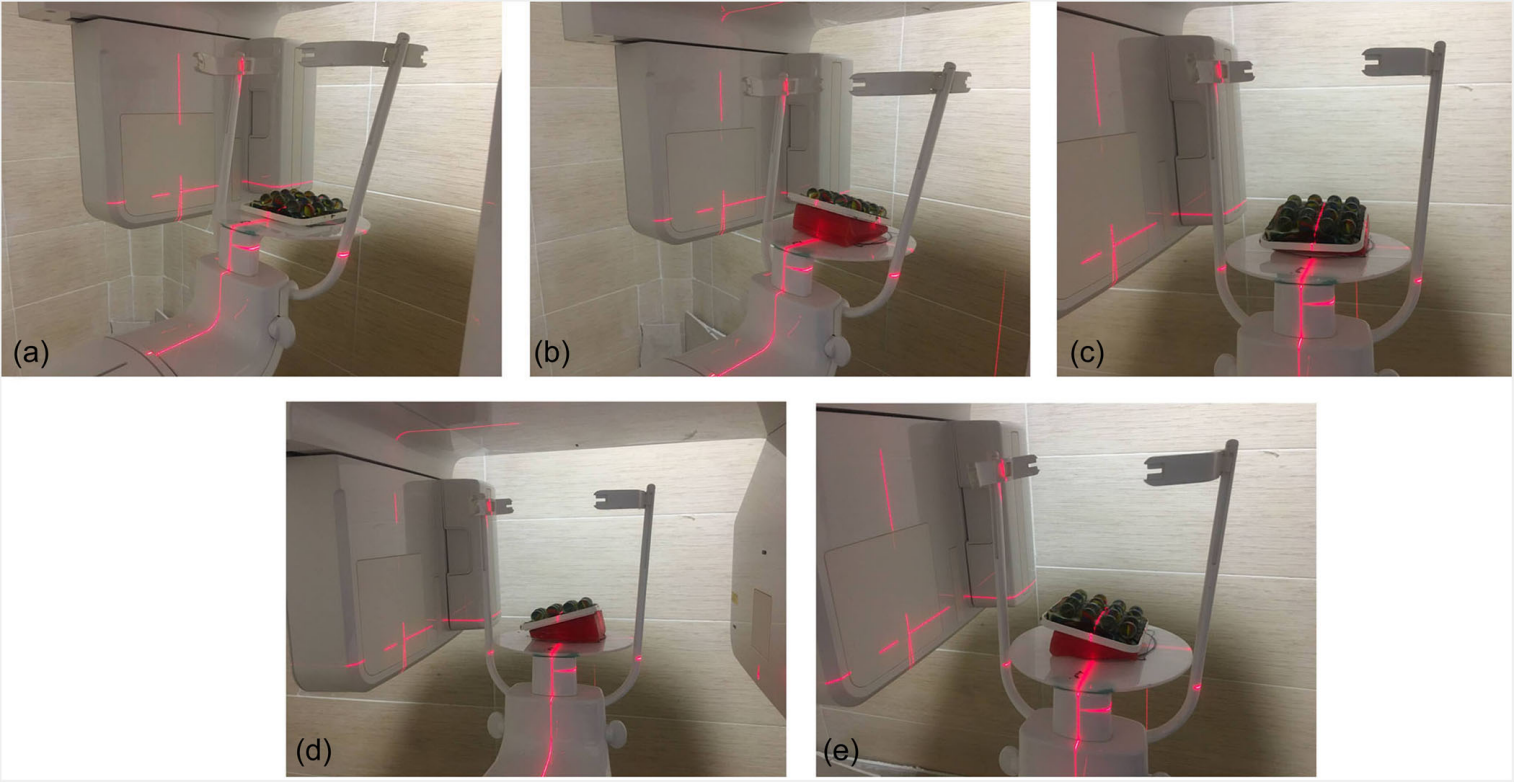
Different scaning positions and stabilization on a flat support on the XMIND Trium CBCT scanner. (a) neutral, (b) 20° tilt to up, (c) 20° tilt to down, (d) 20° tilt to right, (e) 20° tilt to left. CBCT, Cone‐beam computed tomography.

**Figure 2:**
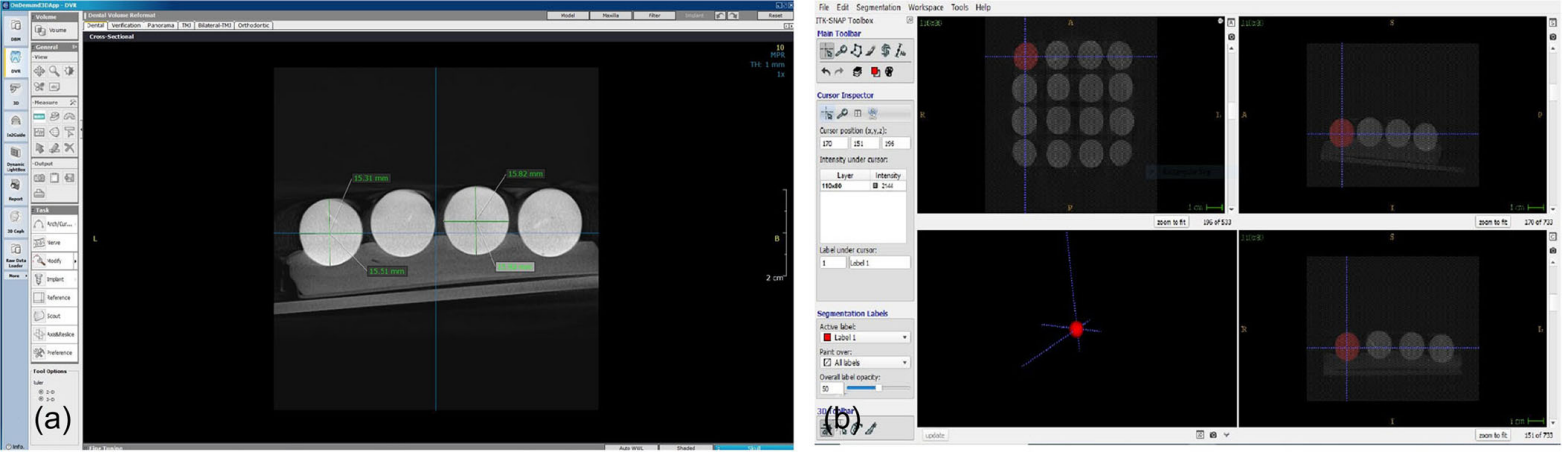
(a) Measurement of horizontal and vertical dimensions in CBCT axial sections in ondemand software. (b) Measurement of ball volume in itk‐snap software. CBCT, Cone‐beam computed tomography.

This study was approved by the ethics committee of Babol University of Medical Sciences (IR.MUBABOL.REC.1400.255). Sixteen glass balls (Zivaralat, Tehran, Iran) with a mean diameter of 15.99 ± 0.34 mm as measured by a digital caliper (Guanglu) were used in this in vitro, experimental study. The glass balls were fixed by embedding in a mixture of paraffin and wax (Aria) in a square‐shaped container. Modeling wax (Cavex) was used to fabricate two inclined planes with 10° and 20° angles. The container with embedded glass balls was scanned first in neutral position (N) (Figure 1a) and then by using the 10° inclined plane with a right tilt (R10°), left tilt (L10°), upward tilt (U10°), and downward tilt (D10°) (4 scans). The same procedures were repeated using the inclined plane with 20° angle (4 scans) (Figure 1b–e). All CBCT scans were obtained using XMIND TRIUM CBCT scanner (Acteon) with the exposure settings of 90 kVp, 10 mA, 8 × 11 cm field of view, 150 µm voxel size, and high resolution. The CBCT scans were saved in DICOM format, and processed using OnDemand 3D Dental software (CyberMed Co). Next, the vertical and horizontal diameters of each ball were separately measured on axial CBCT sections by the electronic ruler feature of the software and by using the reference lines (Figure 2a). Volumetric measurements were made by ITK‐Snap 3.8.0 software (Cognitica) using the segmentation technique (Figure 2b). All measurements were made by one examiner (a postgraduate student of oral and maxillofacial radiology) under the supervision of an oral and maxillofacial radiologist. All linear and volumetric measurements were repeated by the same examiner after 1 month.

Data were analyzed using SPSS version 22 (SPSS Inc). The reproducibility of linear and volumetric measurements made in different positions (N, U10°, U20°, R10°, R20°, L10°, L20°, D10°, and D20°) was analyzed by calculating the Cronbach's *α*. Also, paired t‐test was used to analyze the difference in volumetric and linear measurements of the balls in the anterior and posterior halves of the scans in neutral position versus the upward and downward tilts, and the right and left halves of the scans in neutral position versus the right and left tilts.

## 3 RESULTS

The Cronbach's *α* was found to be .982 and .933 for volumetric and linear measurements, respectively, indicating high reproducibility of the measurements. The mean difference in diameter of the balls in D10°, D20°, L10°, L20°, R10°, R20°, U10°, and U20° positions versus the neutral position was also evaluated (Table 1); the maximum mean difference was 0.32 ± 0.17 mm (20° downward tilt). Also, the difference in the mean linear measurements of the balls in the anterior (8) and posterior (8) halves of the scans in neutral position versus the upward and downward tilts, and balls in the left (8) and right (8) sides of the scans in neutral position versus the left and right tilts was analyzed. The results are presented in Table 2. As shown, the maximum mean difference was 0.29 ± 0.17 mm (20° right tilt). Since in linear measurements, a 2‐mm margin should be considered as safety zone (White & Pharoah, 2019) the obtained values indicated no significant difference in dimensional measurements made in different positions.

**Table 1:** Mean difference in measured ball diameters in different positions compared with neutral position.

Positions			
Mean ± SD* (mm)		MD* ±SD (mm)	95% CI* (mm)
Neutral 15.70 ± 0.31	D10°*	0.25 ± 0.28	0.01–0.39
	15.45 ± 0.31		
	D20°*	0.32 ± 0.27	0.18–0.47
	15.37 ± 0.33		
	L10°*	0.11 ± 0.17	0.02–0.20
	15.59 ± 0.25		
	L20°*	0.23 ± 0.24	0.1–0.36
	15.47 ± 0.33		
	R10°*	0.04 ± 0.17	–0.03–0.11
	15.65 ± 0.25		
	R20°*	0.28 ± 0.20	0.17–0.38
	15.42 ± 0.32		
	U10°*	0.16 ± 0.27	0.02–0.31
	15.54 ± 0.28		
	U20°*	0.15 ± 0.23	0.02–0.27
	15.55 ± 0.21		

Abbreviations: CI, confidence interval; D10°, 10° downward tilt; D20°, 20° downward tilt; L10°, 10° left tilt; L20°, 20° left tilt; MD, mean difference; R10°, 10° right tilt; R20°, 20° right tilt; SD, standard deviation; U10°, 10° upward tilt; U20°, 20° upward tilt.

**Table 2:** Mean difference in measured ball diameters in different halves (right, left, anterior, and posterior) of the scans in different positions compared with neutral position in the respective half.

Scan															
Right half				Left half				Anterior half				Posterior half			
Position mean ± SD* (mm)		MD* ± SD (mm)	CI* 95%	Position mean ± SD (mm)		MD±SD (mm)	CI 95% (mm)	Position mean ± SD (mm)		MD ± SD (mm)	CI 95% (mm)	Position mean ± SD (mm)		MD ± SD (mm)	CI 95% (mm)
Neutral 15.64 ± 0.37	L10°*	0.13 ± 0.22	–0.05–0.31	Neutral 15.75 ± 0.25	L10°	0.09 ± 0.12	–0.01–0.19	Neutral 15.76 ± 0.37	D10°*	0.23 ± 0.43	–0.11–0.59	Neutral 15.63 ± 0.25	D10°	0.09 ± 0.19	–0.05–0.25
	15.51 ± 0.27				15.66 ± 0.23				15.53 ± 0.43				15.54 ± 0.30		
	L20°*	0.26 ± 0.26	0.04–0.48		L20°	0.20 ± 0.22	0.01–0.38		D20°*	0.25 ± 0.38	–0.05–0.57		D20°	0.19 ± 0.15	0.07–0.31
	15.38 ± 0.30				15.55 ± 0.35				15.51 ± 0.38				15.44 ± 0.33		
	R10°*	0.04 ± 0.18	–0.11–0.20		R10°	0.04 ± 0.16	–0.08–0.17		U10°*	–0.02 ± 0.35	–0.30–0.27		U10°	0.01 ± 0.21	–0.16–0.19
	15.60 ± 0.30				15.71 ± 0.21				15.79 ± 0.28				15.61 ± 0.34		
	R20°*	0.26 ± 0.23	0.06–0.45		R20°	0.29 ± 0.17	0.15–0.43		U20°*	0.03 ± 0.20	–0.12–0.19		U20°	–0.00 ± 0.24	–0.20–0.18
	15.39 ± 0.36				15.45 ± 0.31				15.74 ± 0.34				15.65 ± 0.24		

Abbreviations: CI, confidence interval; D10°, 10° downward tilt; D20°, 20° downward tilt; L10°, 10° left tilt; L20°, 20° left tilt; MD, mean difference; R10°, 10° right tilt; R20°, 20° right tilt; SD, standard deviation; U10°, 10° upward tilt; U20°, 20° upward tilt.

Table 3 presents the difference in the mean volumetric measurements of the balls in D10°, D20°, L10°, L20°, R10°, R20°, U10°, and U20° positions compared with the neutral position. As shown, the maximum mean difference was 11.81 ± 42.89 mm^3^ (20° right tilt).

**Table 3:** Mean difference in volumetric measurements of the balls in different positions compared with neutral position.

Positions			
Mean ± SD* (mm³)		MD* ± SD (mm³)	CI* 95% (mm³)
Neutral 2076.31 ± 91.64	D10°*	6.88 ± 54.99	–22.43–36.18
	2069.44 ± 102.93		
	D20°*	3.50 ± 46.61	–21.34–28.34
	2072.81 ± 96.63		
	L10°*	8 ± 39.06	–12.82–28.82
	2068.31 ± 92.80		
	L20°*	1.63 ± 43.92	–21.78–25.03
	2074.69 ± 89.18		
	R10°*	2.13 ± 38.30	–18.27–22.52
	2074.19 ± 86.19		
	R20°*	11.81 ± 42.89	–11.04–34.66
	2064.50 ± 92.94		
	U10°*	0.75 ± 20.42	–10.13–11.63
	2075.56 ± 80.56		
	U20°*	2.75 ± 34.90	–15.85–21.35
	2073.56 ± 77.74		

Abbreviations: CI, confidence interval; D10°, 10° downward tilt; D20°, 20° downward tilt; L10°, 10° left tilt; L20°, 20° left tilt; MD, mean difference; R10°, 10° right tilt; R20°, 20° right tilt; SD, standard deviation; U10°, 10° upward tilt; U20°, 20° upward tilt.

Table 4 presents the mean difference in volumetric measurements of the balls in the anterior (8) and posterior (8) halves of the scans in neutral position versus the upward and downward tilts, and balls in the left (8) and right (8) sides of the scans in neutral position compared with left and right tilts. As indicated, maximum mean difference was 30.50 ± 31.17 mm^3^ (10° right tilt in the left side of the scan), indicating that different positions had no significant effect on volumetric measurements.

**Table 4:** Mean difference in volumetric measurements of the balls in different halves (right, left, upper, and lower) of the scans in different positions compared with neutral position in the respective half.

Scan															
Right half				Left half				Anterior half				Posterior half			
Position mean ± SD*(mm³)		MD* ± SD (mm³)	CI* 95% (mm³)	Position mean ± SD (mm³)		MD ± SD (mm³)	CI 95% (mm³)	Position mean ± SD (mm³)		MD±SD (mm³)	CI 95% (mm³)	Position mean ± SD (mm³)		MD ± SD (mm³)	CI 95% (mm³)
Neutral 2035.75 ± 107.37	L10°*	18.75 ± 48.91	–22.14–59.64	Neutral 2116.88 ± 52	L10°	–2.75 ± 24.78	–23.46–17.91	Nutral 2083.5 ± 117.77	D10°*	–23.61 ± 39.10	–57.06–9.81	Neutral 2069.13 ± 73.37	D10°	37.38 ± 52.47	–6.49–81.24
	2017 ± 95.70				2119.63 ± 57.25				2107.13 ± 112.55				2031.75 ± 82.39		
	L20°*	19.88 ± 54.36	–25.57–65.32		L20°	–16.63 ± 20.42	–33.70–0.45		D20°*	–26.11 ± 40.14	–59.68–7.43		D20°	33.13 ± 32.22	6.18–60.07
	2015.8 ± 78.04				2133.5 ± 55.2				2109.63 ± 110.56				2036 ± 68.46		
	R10°*	–26.25 ± 18.12	–41.40–(–11.10)		R10°	30.5 ± 31.17	4.44–56.56		U10°*	4.63 ± 26.40	–17.45–26.70		U10°	–3.13 ± 12.72	–13.76–7.51
	2062 ± 103.06				2086.38 ± 70.42				2078.88 ± 95.28				2072.25 ± 69.32		
	R20°*	2.13 ± 47.42	–37.52–41.77		R20°	21.5 ± 38.44	–10.64–53.64		U20°*	11.13 ± 36.58	–19.440–41.70		U20°	–5.63 ± 33.34	–33.50–22.25
	2033.6 ± 110.51				2095.38 ± 64.16				2072.38 ± 91.94				2074.75 ± 67.04		

Abbreviations: CI, confidence interval; D10°, 10° downward tilt; D20°, 20° downward tilt; L10°, 10° left tilt; L20°, 20° left tilt; MD, mean difference; R10°, 10° right tilt; R20°, 20° right tilt; SD, standard deviation; U10°, 10° upward tilt; U20°, 20° upward tilt.

The intraobserver agreement for volumetric and linear measurements was found to be 0.983 and 0.887, respectively (*p* < .001).

## 4 DISCUSSION

Having accurate and reliable measurement becomes very important to assess the bone for implant placement, as well as the amount of bone graft required for maxillofacial surgeries. numerous authers have evaluated the accuracy of CBCT images, but none of them evaluated the influence of different head positions on volumetric measurements.

The present study evaluated the mean difference in linear measurements made on CBCT scans in different positions (D10°, D20°, L10°, L20°, R10°, R20°, U10°, and U20°) compared with neutral position, which revealed no significant dimensional difference in different positions. Also, volumetric measurements were made using ITK‐Snap software. The reliability and validity of this software have been previously confirmed in the literature (Almuzian et al., 2018; Gomes et al., 2020). In volumetric measurements made by this software, the maximum difference with neutral position was 1%, which is negligible. Thus, it appears that volumetric measurements made in different positions had no significant difference with those made in neutral position.

The results of studies carried out by (Adibi et al., 2017; Almeida et al., 2021; Kakumoto et al., 2021; Shokri et al., 2016) regarding the accuracy of linear measurements confirmed the present findings. In line with the present results, Tolentino et al (Tolentino et al., 2018) performed linear measurements on CBCT scans with three different voxel sizes using three different software programs. They observed that the software programs were reliable and accurate in comparison with the gold‐standard physical measurements, and voxel size had no significant effect on the accuracy of linear measurements. Panjnoush et al. (2017) evaluated the accuracy of linear measurements in five different positions (central, left, right, anterior and posterior). Similar to the present study, they reported that object position had no clinically significant effect on the accuracy of longitudinal and horizontal measurements. They added that CBCT can be used as an accurate modality for dimensional measurements of objects in different positions within the field of view. With respect to the accuracy of linear measurements, Costa et al (Costa et al., 2018) reported results similar to the present finding. Their study was the effect of voxel size on linear measurement accuracy. They measured the mediolateral and anteroposterior dimensions of the condyle on CBCT 3D images and observed that a smaller voxel size does not necessarily increase accuracy, And the same accuracy can be obtained with a larger voxel size and less radiation. In our study, the horizontal and vertical dimensions of the balls were measured on the CBCT axial images in same voxel size but different positions and no significant difference was found in linear measurements.

However, Zhao et al. (2021) Sabban et al. (2015) reported different results. Zhao et al. (2021) reported low linear accuracy of CBCT. They measured soft tissue dimensions on 3D views while vertical and horizontal diameters of the balls were measured on axial sections in the present study. Difference between their results and the present findings may be due to the lower accuracy of linear measurements made on 3D views. Unlike the present results, Sabban et al. (2015) found a significant difference in CBCT measurements and the gold standard in vertical dimension in extension position of the head compared with centric position. Difference in the results of the two studies may be attributed to the difference in voxel size since they used a 293‐µm voxel size while in the present study, linear measurements were made on images taken with a smaller voxel size (150 µm), which may affect the accuracy of linear measurements.

With respect to the accuracy of volumetric measurements, García‐Sanz et al. (2017) reported results similar to the present findings. They performed volumetric measurements by using the volumetric measurement tool of Dolphin software, and confirmed that CBCT is an accurate and reproducible modality for volumetric measurements.

Zhou et al. (2015) performed 3D measurements on axial, coronal, and sagittal sections to measure the graft volume required for unilateral cleft palate patients using Simplant software, and compared the values with the gold standard. They reported that CBCT measurements were accurate for calculation of the volume of alveolar defect, and quantification of the required graft volume. Similar results were reported by Sönmez et al. (2018) as well. However, Etemadi Sh et al. (2021) and Dong et al. (2019) reported contrary results. Difference between the results of Etemadi Sh et al. (2021) and the present findings may be attributed to the difference in method of volumetric measurements since they indirectly calculated the volume based on the surface areas computed by the software, which is less accurate than the values reported in the present study, since a software program exclusive for volumetric measurements was used in the present study for direct volumetric measurements.

Dong et al. (2019) found a significant difference in volumetric measurements made by using different voxel sizes. However, in the present study, the same voxel size was used in different positions.

One strength of the present study was evaluation of glass balls with unequal but close diameters, which prevented bias by the examiner. Further studies on higher number of balls are required using different voxel sizes.

Considering the very small difference in linear and volumetric measurements made in different positions, it appears that CBCT is a reliable modality for such measurements in different positions.

The limitation of this pilot study includes small sample size. it is recommended to conduct more studies with larger sample size, larger field of view and different voxel sizes.

## AUTHOR CONTRIBUTIONS

*Study concept and design*: Arash Poursattar‐Bejeh Mir. *Acquisition of data*: Samira Fani. *Analysis and interpretation of data*: Sina Haghanifar. *Drafting of the manuscript*: Samira Fani. *Critical revision of the manuscript for important intellectual content*: Ehsan Moudi. *Statistical analysis*: Seyedali seyedmajidi. *Administrative, technical, and material support*: Ehsan Moudi and Samira Fani. *Study supervision*: Ehsan Moudi.

## CONFLICT OF INTEREST STATEMENT

The authors declare no conflict of interest.

## Data Availability

The data that support the findings of this study are available on request from the corresponding author. The data are not publicly available due to privacy or ethical restrictions.

## References

[cre2732-bib-0001] Adibi, S. , Shahidi, S. , Nikanjam, S. , Paknahad, M. , & Ranjbar, M. (2017). Influence of head position on the CBCT accuracy in assessment of the proximity of the root apices to the inferior alveolar canal. Journal of dentistry (Shiraz, Iran), 18(3), 181–186. 29034272 PMC5634357

[cre2732-bib-0002] Almeida, V. S. M. , Bomfim, R. T. , Sobreira, A. C. R. , Barbosa, I. S. , Leite‐Ribeiro, P. M. , Rubira‐Bullen, I. R. , & Sarmento, V. A. (2021). Linear measurement accuracy of CBCT panoramic reconstructions: Experimental study with dry human mandibles. Oral Radiology, 37(3), 421–426. 10.1007/s11282-020-00477-4 32936399

[cre2732-bib-0003] Almuzian, M. , Ghatam, H. A. , & Al‐Muzian, L. (2018). Assessing the validity of ITK‐SNAP software package in measuring the volume of upper airway spaces secondary to rapid maxillary expansion. Journal of Orthodontic Science, 7, 7. 10.4103/jos.JOS_93_17 29765919 PMC5952232

[cre2732-bib-0004] Costa, A. , Barbosa, B. , Perez‐Gomes, J. , Calle, A. , Santamaria, M. , & Lopes, S. (2018). Influence of voxel size on the accuracy of linear measurements of the condyle in images of cone beam computed tomography: A pilot study. Journal of Clinical and Experimental Dentistry, 10(9),876–882. 10.4317/jced.54500 PMC6203913 30386520

[cre2732-bib-0005] Dong, T. , Xia, L. , Cai, C. , Yuan, L. , Ye, N. , & Fang, B. (2019). Accuracy of in vitro mandibular volumetric measurements from CBCT of different voxel sizes with different segmentation threshold settings. BMC Oral Health, 19(1), 206. 10.1186/s12903-019-0891-5 31484529 PMC6727515

[cre2732-bib-0006] Etemadi Sh, M. , Movahedian Attar, B. , Mehdizadeh, M. , & Tajmiri, G. (2021). Evaluation of the CBCT imaging accuracy in the volumetric assessment of unilateral alveolar cleft. Journal of Stomatology, Oral and Maxillofacial Surgery, 122(4), e1–e5. 10.1016/j.jormas.2021.06.006 34175477

[cre2732-bib-0007] García‐Sanz, V. , Bellot‐Arcís, C. , Hernández, V. , Serrano‐Sánchez, P. , Guarinos, J. , & Paredes‐Gallardo, V. (2017). Accuracy and reliability of cone‐beam computed tomography for linear and volumetric mandibular condyle measurements. A human cadaver study. Scientific Reports, 7(1), 11993. 10.1038/s41598-017-12100-4 28931867 PMC5607232

[cre2732-bib-0008] Gomes, A. F. , Brasil, D. M. , Silva, A. I. V. , Freitas, D. Q. , Haiter‐Neto, F. , & Groppo, F. C. (2020). Accuracy of ITK‐SNAP software for 3D analysis of a non‐regular topography structure. Oral Radiology, 36(2), 183–189. 10.1007/s11282-019-00397-y 31267257

[cre2732-bib-0009] Kakumoto, T. , Barsoum, A. , & Froum, S. J. (2021). Accuracy of cone‐beam computed tomography versus periapical radiography measurements when planning placement of implants in the posterior maxilla: A retrospective study. Compendium of Continuing Education in Dentistry (Jamesburg, N.J.: 1995), 42(7), 1. 34270273

[cre2732-bib-0010] Mupparapu, M. , Shi, K. J. , Lo, A. D. , & Setzer, F. C. (2021). Novel 3D segmentation for reliable volumetric assessment of the nasal airway: A CBCT study. Quintessence International (Berlin, Germany: 1985), 52(2), 154–164. 10.3290/j.qi.a45429 33433081

[cre2732-bib-0011] Panjnoush, M. , Kheirandish, Y. , & Zeini, N. (2017). Effect of spatial position in the field of view on dimensional changes in cone beam computed tomography. Journal of Dentistry (Tehran, Iran), 14(5), 282–291. 29296114 PMC5748456

[cre2732-bib-0012] Rokn, A. R. , Hashemi, K. , Akbari, S. , Kharazifard, M. J. , Barikani, H. , & Panjnoosh, M. (2016). Accuracy of linear measurements using cone beam computed tomography in comparison with clinical measurements. Journal of Dentistry (Tehran, Iran), 13(5), 333–339. 28127327 PMC5250631

[cre2732-bib-0013] Sabban, H. , Mahdian, M. , Dhingra, A. , Lurie, A. G. , & Tadinada, A. (2015). Evaluation of linear measurements of implant sites based on head orientation during acquisition: An ex vivo study using cone‐beam computed tomography. Imaging Science in Dentistry, 45(2), 73–80. 10.5624/isd.2015.45.2.73 26125001 PMC4483623

[cre2732-bib-0014] Shokri, A. , Miresmaeili, A. , Farhadian, N. , Falah‐Kooshki, S. , Amini, P. , & Mollaie, N. (2016). Effect of head position on maxillofacial transverse measurements made on the skull and cone beam computed tomography scans. Brazilian Dental Journal, 27(5), 604–608. 10.1590/0103-6440201601166 27982242

[cre2732-bib-0015] Sönmez, G. , Koç, C. , & Kamburoğlu, K. (2018). Accuracy of linear and volumetric measurements of artificial ERR cavities by using CBCT images obtained at 4 different voxel sizes and measured by using 4 different software: An ex vivo research. Dentomaxillofacial Radiology, 47(8), 20170325. 10.1259/dmfr.20170325 29851352 PMC6326391

[cre2732-bib-0016] Tofangchiha, M. , Bardal, R. , Hosseini, M. S. , & Najafi, K. (2016). Effect of mandibular plane changes on angular measurements in cone beam computed tomography. Journal of Inflammatory Diseases, 19(6), 49–43.

[cre2732-bib-0017] Tolentino, E. S. , Yamashita, F. , de Albuquerque, S. , Walewski, L. , Iwaki, L. C. V. , Takeshita, W. , & Silva, M. (2018). Reliability and accuracy of linear measurements in cone‐beam computed tomography using different software programs and voxel sizes. Journal of Conservative Dentistry, 21(6), 607–612. 10.4103/JCD.JCD_314_18 30546204 PMC6249944

[cre2732-bib-0018] White, S. C. , & Pharoah, M. J. (2019). White and Pharoah's oral radiology: Principles and interpretation. Elsevier Health Sciences.

[cre2732-bib-0019] Zhao, Z. , Xie, L. , Cao, D. , Izadikhah, I. , Gao, P. , Zhao, Y. , & Yan, B. (2021). Accuracy of three‐dimensional photogrammetry and cone beam computed tomography based on linear measurements in patients with facial deformities. Dentomaxillofacial Radiology, 50(2), 20200001. 10.1259/dmfr.20200001 32791014 PMC7860960

[cre2732-bib-0020] Zhou, W. , Xu, Y. , Jiang, H. , Wan, L. , & Du, Y. (2015). Accurate evaluation of cone‐beam computed tomography to volumetrically assess bone grafting in alveolar cleft patients. Journal of Craniofacial Surgery, 26(6), e535–e539. 10.1097/SCS.0000000000002034 26355988

